# Monophosphoryl-Lipid A (MPLA) is an Efficacious Adjuvant for Inactivated Rabies Vaccines

**DOI:** 10.3390/v11121118

**Published:** 2019-12-03

**Authors:** Chen Chen, Chengguang Zhang, Ruiming Li, Zongmei Wang, Yueming Yuan, Haoqi Li, Zhenfang Fu, Ming Zhou, Ling Zhao

**Affiliations:** 1 State Key Laboratory of Agricultural Microbiology, Huazhong Agricultural University, Wuhan 430070, China; ccatwh@163.com (C.C.); zhangcg@webmail.hzau.edu.cn (C.Z.); mhming616@163.com (R.L.); 18855170704@163.com (Z.W.); 18086188238@163.com (Y.Y.); lhqgsts@163.com (H.L.); zhenfu@uga.edu (Z.F.); mikchail@163.com (M.Z.); 2 College of Veterinary Medicine, Huazhong Agricultural University, Wuhan 430070, China; 3 Key Laboratory of Preventive Veterinary Medicine of Hubei Province, Huazhong Agricultural University, Wuhan 430070, China; 4 Department of Pathology, College of Veterinary Medicine, University of Georgia, Athens, GA 30602, USA

**Keywords:** rabies virus, MPLA, inactivated vaccine, humoral immunity

## Abstract

Rabies, as one of the most threatening zoonoses in the world, causes a fatal central nervous system (CNS) disease. So far, vaccination with rabies vaccines has been the most effective measure to prevent and control this disease. At present, inactivated rabies vaccines are widely used in humans and domestic animals. However, humoral immune responses induced by inactivated rabies vaccines are relatively low and multiple shots are required to achieve protective immunity. Supplementation with an adjuvant is a practical way to improve the immunogenicity of inactivated rabies vaccines. In this study, we found that monophosphoryl-lipid A (MPLA), a well-known TLR4 agonist, could significantly promote the maturation of bone marrow-derived dendritic cells (BMDC) through a TLR4-dependent pathway in vitro and the maturation of conventional DCs (cDCs) in vivo. We also found that MPLA, serving as an adjuvant for inactivated rabies vaccines, could significantly facilitate the generation of T follicular helper (Tfh) cells, germinal center (GC) B cells, and plasma cells (PCs), consequently enhancing the production of RABV-specific total-IgG, IgG2a, IgG2b, and the virus-neutralizing antibodies (VNAs). Furthermore, MPLA could increase the survival ratio of mice challenged with virulent RABV. In conclusion, our results demonstrate that MPLA serving as an adjuvant enhances the intensity of humoral immune responses by activating the cDC–Tfh–GC B axis. Our findings will contribute to the improvement of the efficiency of traditional rabies vaccines.

## 1. Introduction

Rabies virus (RABV), a single negative-strand RNA virus, belonging to the *Lyssavirus* genus within the *Rhabdoviridae* family, is still responsible for 59,000–61,000 human deaths annually, mostly in developing countries [1,2,3]. The RABV genome encodes five structural proteins, including nucleocapsid protein (N), phosphoprotein (P), matrix protein (M), glycoprotein (G), and large polymerase (L) [4]. After auto-cleaving the first 19 amino acids (aa), defined as the signal peptide (sp) of the G protein precursor, the mature G protein (1–505 aa), which is comprised of the ectodomain at the 5′ end (et, 1–439 aa), the transmembrane domain (tm, 440–461 aa) and the cytoplasmic tail (ct, 462–505 aa), accesses the virion surface [5,6]. Importantly, the G protein is the only protein on the virion surface, and it is mainly responsible for the interaction with receptors expressed on the cell surface [7,8]. In addition, the G protein is the only protein to induce virus-neutralizing antibodies (VNA) [4].

Pre-exposure prophylaxis (PrEP) and post-exposure prophylaxis (PEP) are the main methods for rabies prevention and control. In recent years, recombinant virus vectors such as poxviruses, paramyxoviruses and adenovirus have become promising for research and development of novel rabies vaccines [9,10,11]. Nevertheless, both attenuated RABV and the recombinant virus may reserve potential virulence, which may become a major obstacle for acquiring licenses in many countries. Inactivated vaccines are still widely used in human and domestic animals due to their high safety. However, the efficiency of inactivated RABV vaccines is relatively low compared with that of live attenuated vaccines and more than one shot is required to achieve protective immunity. Supplementation with adjuvants is a practical strategy to boost the immunogenicity of inactivated RABV vaccines. So far, the aluminum adjuvant for inactivated rabies vaccine is under pre-clinical study [12], and the PIKA (a synthetic double-stranded RNA analogue) adjuvant has progressed to a phase II trial in healthy adults [13]. PIKA-containing rabies vaccine is more effective in preventing rabies due to its ability to activate the Toll-like receptor 3 (TLR3) pathway compared to adjuvant-free vaccines [14].

TLRs are promising immune sensors, and play a crucial role in defending against pathogenic microbial infection [15,16]. Several previous studies reported that, as an innate immune sensor, TLR4 recognizes both microbial and endogenous ligands, and initiates an immediate immune response to them [17,18]. Our previous study has indicated that the high mobility group box 1 protein (HMGB1), well-known as a TLR4 ligand, could improve humoral immunity through dendritic cell (DC) activation [19]. Furthermore, the widely acknowledged TLR4 agonist, monophosphoryl-lipid A (MPLA), could induce a strong type-1 CD4T helper cell (Th1) immune response, which plays a critical role in affinity maturation of antibodies and has been recently licensed as an adjuvant of the human papilloma virus (HPV) vaccine in Europe and the USA [20,21]. However, RABV-specific VNA and the protective effect of immunization with rabies vaccines supplemented with MPLA have not been investigated yet. In this study, the effect of MPLA as an adjuvant of inactivated rabies vaccine was evaluated in a mouse model. Our results demonstrate that MPLA could improve RABV-specific VNA and protect against virulent RABV challenge.

## 2. Materials and Methods

### 2.1. Cells and Viruses

RABV vaccine strain LBNSE carrying two mutants in the G protein at amino acid position 194 and 333 were generated from the SAD L16 cDNA clone, as described previously [22]. Recombinant (r) RABVs were rescued from B7GG cells (kindly provided by Dr. Gang Cao at Huazhong Agricultural University, Wuhan, China) and amplified in BSR cells (kindly provided by Dr. Bernhard Dietzschold at Thomas Jefferson University, Philadelphia, USA) [23]. A dog-derived RABV wild type strain, DRV-Mexico, was isolated from a human patient and propagated in the brains of newborn mice [24,25]. RABV titers and RABV-specific neutralizing antibodies were titrated in BSR cells. Both these cell lines were grown in Dulbecco’s modified Eagle’s medium (DMEM, cat. no. 11965-092) purchased from Thermo Fisher Scientific Inc. (Waltham, MA, USA). Wild type (WT) or TLR4 knock-out (TLR4^−/−^) mice-derived bone marrow-derived dendritic cells (BMDC) were maintained in RMPI-1640 medium supplemented with 20 ng/mL of granulocyte-macrophage colony stimulating factor (GM-CSF, cat. no. 315-03-20) and 10 ng/mL of interleukin-4 (IL-4, cat. no. 214-14-20) purchased from Peprotech Inc. (Rocky Hill, NJ, USA), and BMDC cells were utilized to detect TLR4-dependent cell activation. Complete culture medium was supplemented with 10% (*v*/*v*) fetal bovine/calf serum (FBS, cat. no. 10099141) purchased from Thermo Fisher Scientific Inc. (Waltham, MA, USA) and 1% (*v*/*v*) penicillin/streptomycin (cat. no. C0222) purchased from Beyotime Biotechnology Co., Ltd. (Shanghai, CHN). All cells were maintained in an incubator with 5% CO_2_ at 37 °C.

### 2.2. Antibodies

Various antibodies with direct-labelled fluorescein for different cell markers were utilized to analyze several types of immune cells. FITC anti-mouse CD11c antibody (cat. no. 117306), APC anti-mouse CD80 antibody (cat. no. 104714), PE anti-mouse CD86 antibody (cat. no. 105008) and PE/Cy7 anti-mouse I-A/I-E (MHCII) antibody (cat. no. 107630) were used to analyze the activation/maturation of BMDC in vitro and conventional DC (cDC) in vivo. FITC anti-mouse CD4 antibody (cat. no. 100510), APC anti-mouse CD185 (CXCR5) antibody (cat. no. 145506), and PE anti-mouse CD279 (PD1) antibody (cat. no. 135206) were utilized to analyze the number of T follicular helper (Tfh) cells in inguinal lymph nodes (LNs) [23]. FITC anti-mouse/human CD45R/B220 antibody (cat. no. 103206), 647 anti-mouse/human GL7 antibody (cat. no. 144606), and PE anti-mouse CD95 antibody (cat. no. 554295) were applied to analyze the number of germinal center B (GC B) cells in LNs [26]. FITC anti-mouse/human CD45R/B220 antibody (cat. no. 103206) and APC anti-mouse CD138 (Syndecan-1) antibody (cat. no. 142506) were used to analyze the number of plasma cells (PCs) in bone marrows (BMs). All of these antibodies were purchased from BioLegend, Inc. (San Diego, CA, USA) or BD Bioscience, Inc. (Franklin Lakes, NJ, USA).

### 2.3. Animals

Female ICR and C57BL/6 mice were obtained from the Center for Disease Control and Prevention of Hubei Province, China. The C57BL/10 wild type (WT) mice and TLR4 knock-out (TLR4^−/−^) mice in C57BL/10 background were purchased from GemPharmatech Co., Ltd. (Nanjing, CHN). All mice were housed in the Animal Facility at Huazhong Agricultural University. For the analysis of flow cytometry, three groups (*n* = 5/group) of 6-week-old female C57BL/6 mice were respectively inoculated with 100 μL DMEM or beta-propiolactone (BPL)-inactivated LBNSE (1 × 10^7^ FFU) or BPL-inactivated LBNSE (1 × 10^7^ FFU) supplemented with 20 μg MPLA (cat. no. vac-mpls) purchased from Invivogen Inc. (San Diego, CA, USA) via IM injection. The inguinal LNs were collected on day 7 and 14 (day 3 and 6 were appropriate for dendritic cells analysis) after immunization and prepared for flow cytometry. To measure RABV-specific VNA, total-immunoglobulin (Ig) G and Ig subtypes including IgG1, IgG2a and IgG2b, in three groups of 6-week-old female ICR mice (*n* = 10/group), were inoculated with 100 μL DMEM, BPL-inactivated LBNSE (1 × 10^7^ FFU), or BPL-inactivated LBNSE (1 × 10^7^ FFU) supplemented with 20 μg MPLA via IM injection, respectively. The sera of mice were harvested every week until 8 weeks after immunization. For evaluating the protection of rabies vaccines, all three groups of mice were challenged with 100 × LD_50_ DRV-Mexico at the eighth week after immunization and monitored for another 3 weeks. Mice that appeared moribund or that had lost more than 25% of their starting body weight were humanely euthanized with CO_2_.

### 2.4. Virus Titration

Virus titer of RABV was measured by using direct immunofluorescence assays in BSR cells, as described previously [26,27]. Briefly, viruses were 10-fold serially diluted and added into 96-well plates, then BSR cells (2 × 10^4^ cells/well) were incubated with serially diluted RABV at 37 °C for 48 h. After incubation, the culture medium was discarded, and the adherent cells were fixed with 80% ice-cold acetone at −20 °C for 1 h. After 3 washes with PBS, the cells were stained with FITC-conjugated anti-RABV N protein antibodies for 1 h at 37 °C. After 3 washes with PBS, antigen-positive fluorescent foci on the cells were counted under an Olympus IX51 fluorescence microscope (Olympus, Tokyo, JPN), and virus titers were calculated as fluorescent focus units per ml. All titrations were carried out in quadruplicate.

### 2.5. Inactivation of RABV

The supernatant of LBNSE-infected BSR cells was harvested and centrifuged at 10,000 rpm for removal of cell debris. The beta-propiolactone (BPL, cat. no. P9620) was purchased from Sigma-Aldrich Co., Ltd. (Darmstadt, GER), and used as an inactivator for RABV as described previously [28,29]. The 1 × 10^8^ FFU/ml LBNSE in the cleaned medium was inactivated with 0.025% (*v*/*v*) BPL at 4 °C for 24 h. The residual BPL was hydrolyzed in a water bath at 37 °C for 2 h. The inactivated LBNSE or LBNSE mixed with 20 μg MPLA was used for mouse immunization.

### 2.6. Preparation of Bone Marrow-Derived DCs (BMDCs)

BMDCs were differentiated and harvested, as described previously [19,30]. Briefly, C57BL/6 mice were euthanized, and BM was obtained from the tibia and femur. The BM cells were filtered through a plastic 40-μm mesh and resuspended in a complete medium; namely, RPMI-1640 supplemented with glutamine, penicillin/streptomycin, and 10% heat-inactivated FBS. Subsequently, 2 mL of medium containing 1 × 10^6^ BMs cells, 20 ng/mL GM-CSF and 10 ng/mL IL-4 was put into one well of 6-well plates. Half of the medium was removed on day 2. New medium supplemented with GM-CSF (2×, 40 ng/mL) and IL-4 (2×, 20 ng/mL) was warmed to reach 37 °C, and then was added into the well. On day 6, the non-adherent cells in the culture supernatant and semi-adherent cells were collected. BMDC cells were harvested and washed once in 10 mL of RPMI-1640 medium, and the treated BMDC cells were used as the raw materials for experiments.

### 2.7. Titration of RABV-Specific VNA

RABV-specific VNA titers were measured by the fluorescent antibody virus neutralization (FAVN) test in BSR cells, as described previously [31]. Briefly, the serum of mice was separated and inactivated for 30 min at 56 °C, and then, 100 μL of DMEM was added into a 96-well plate, and 50 μL of inactivated serum was added into the first column and serially diluted three-fold. After dilution, the serum was neutralized with 100 FFU of the rabies challenge virus (CVS-11) per well for 1 h at 37 °C. After incubation, BSR cells at 2 × 10^4^ cells per well were added into the neutralization medium in the 96-well plates. After incubation for 72 h at 37 °C in 5% CO_2_, cells were then fixed with 80% ice-cold acetone at −20 °C for 30 min and stained with FITC-conjugated RABV N-protein antibodies at 37 °C for 1 h. Fluorescence was observed under an Olympus IX51 fluorescence microscope (Olympus, JPN). The fluorescence values of measured serum were compared with those of a reference serum obtained from the National Institute for Biological Standards and Control, Hertfordshire, UK, and the results were normalized and quantified in international units per ml.

### 2.8. Measurement of RABV-Specific Total-IgG and Immunoglobulin Isotyping

RABV-specific total-IgG in serum was determined by enzyme-linked immunosorbent assay (ELISA), as described previously [23]. Sera from mice were collected and inactivated at 56 °C for 30 min. In brief, 100 μL of coating buffer (5 mM Na_2_CO_3_, pH 9.6) containing 500 ng of purified-RABV per well was added to the ELISA plate and was incubated overnight at 4 °C. The next day, the plate was washed three times with PBST (0.5% tween-80, *w*/*v*), and blocked with PBST containing 5% (*w*/*v*) low-fat milk for 6 h at 4 °C. The serum was then diluted in PBST containing 5% low-fat milk at 1:8000 for total-IgG, at 1:600 for IgG2a, IgG2b and IgG1. Afterwards, 100 μL of the diluted serum was added to the plates and was incubated for 1.5 h at 37 °C. After incubation, the medium in the plates was discarded, and the plates were washed three times with PBST and then were incubated with 100 μL of horseradish peroxidase (HRP)-conjugated goat anti-mouse IgG (cat. no. BA1051, Boster, Wuhan, CHN) diluted at 1:10,000 for 1 h at 37 °C, or incubated with HRP-conjugated second-antibodies from Immunoglobulin Isotyping Kit (cat. no. BF06004, BioDrago, Beijing, CHN). After incubation, the plates were rewashed three times with PBST, and a chromogenic reaction in HRP-labeled plates was performed by using 100 μL of tetra-methyl-benzidine (TMB) substrate (cat. no. AR1104, Boster, Wuhan, CHN) for 25 min, followed by the addition of 50 μL of 2 M H_2_SO_4_ to stop the reaction. The optical density (450 nm) value was measured using a Spectra Max 190 spectrophotometer (Molecular Devices, Sunnyvale, CA, USA).

### 2.9. Flow Cytometric Analysis

Immune cells in the inguinal LNs and BM were analyzed by using flow cytometry. Briefly, the LNs and BM of mice were collected, and solid tissues were carefully ground in pre-cooled PBS (pH 7.4). The cells were resuspended in PBS containing 0.2% BSA, *w*/*v*, and transferred into a tube through a 40-μm nylon filter, then centrifuged, and washed with PBS containing 0.2% BSA. Red blood cells were removed by using lysis buffer (cat. no. 555899, BD Biosciences Inc., Franklin Lakes, NJ, USA). After washing twice, the single suspended cells in PBS containing 0.2% BSA were counted. In total, 1 × 10^6^ cells were stained for flow cytometric antibody analysis. After incubation for 30 min at 4 °C, the cells were washed twice in PBS (containing 0.2% BSA). Finally, stained cells were analyzed using a BD FACSVerse (BD Biosciences Inc., Franklin Lakes, NJ, USA).

### 2.10. Statistics

We used GraphPad Prism 6.0 (GraphPad Software, Inc., San Diego, CA) to perform the statistical analysis. Results are expressed as mean ± standard deviation (SD) or mean ± standard error of the mean (SEM). For analyses of survival data, the log-rank test was used. The significance of the differences between groups was evaluated by one-way analysis of variance followed by Tukey’s post hoc test or student’s *t*-test. *p* values of ≤ 0.05 (*), ≤ 0.01 (**), and ≤ 0.001 (***) between different groups were regarded as significant, highly significant, and very highly significant, respectively.

### 2.11. Ethical Approval

This study was carried out in strict accordance with the recommendations in the guide for the care and use of laboratory animals of the Ministry of Science and Technology of China. All animal experiments were performed in accordance with Chinese law and were approved by the Scientific Ethics Committee of Huazhong Agricultural University with the permit number of HZAUMO-2018-039 on 8 April 2018.

## 3. Results

### 3.1. MPLA Facilitates the Maturation of BMDC Via a TLR4-Dependent Pathway

The ability of MPLA to modulate immune responses has been well studied before [20,32]. Activation of dendritic cells (DC) was reported to contribute to humoral immune responses induced by live rabies vaccine [19,31,33]. In order to evaluate the ability of MPLA to activate DCs in vitro, bone marrow-derived dendritic cells (BMDC) from wild type (WT) mice and TLR4 knock-out (TLR4^−/−^) mice were cultured for 6 days, and then harvested with the above-mentioned method. The 2 × 10^5^ BMDC cells derived from WT mice or TLR4^−/−^ mice were transferred into the 12-well plates and treated with MPLA (100 μL of 1 μg), BPL-inactivated LBNSE (100 μL of 1 × 10^7^ FFU), and DMEM (100 μL), respectively. At 24 h post-treatment, non-adherent cells in the culture supernatant and adherent cells at the bottom of the plate were collected, washed, and stained for identifying CD11c and CD86 (costimulatory molecules), both of which served as BMDC maturation markers. 

**Figure 1:**
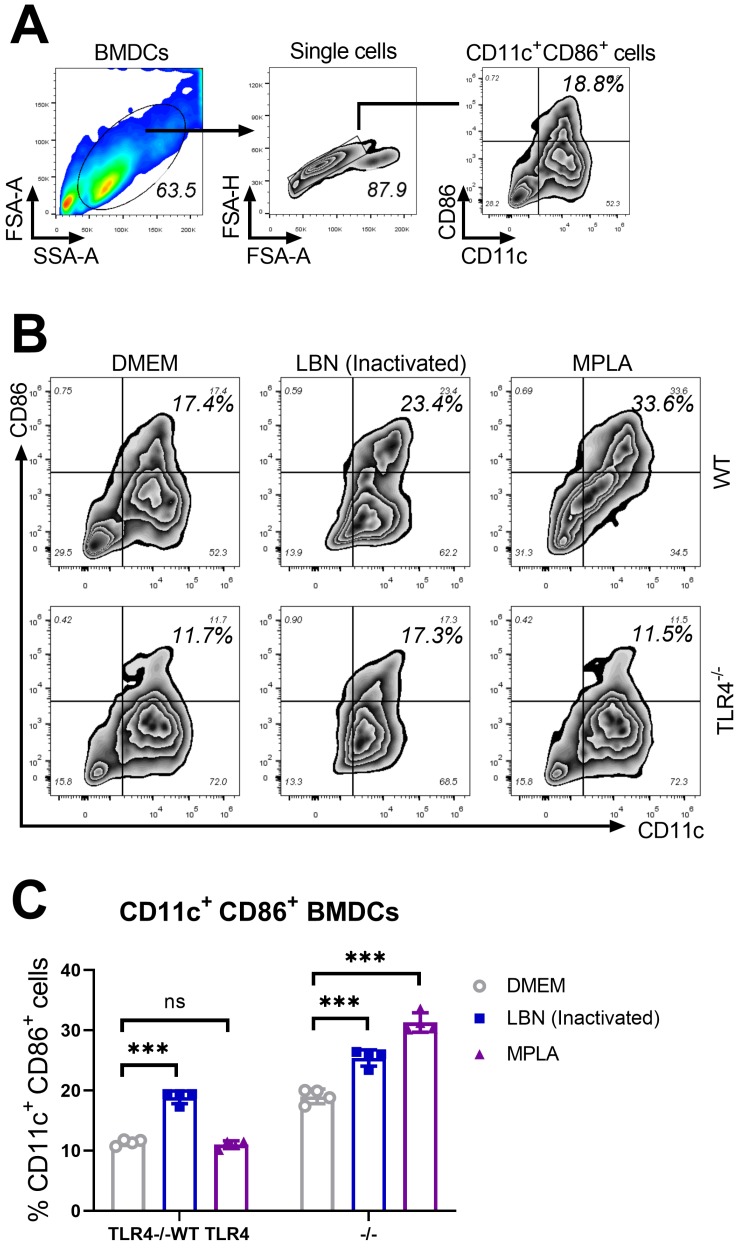
MPLA promotes the maturation of BMDCs via a TLR4-dependent pathway. Wild type (WT) and TLR4 knock-out (TLR4^−/−^) mice were euthanized, and their BM cells were collected from femurs and tibias for the culture of BMDCs in the presence of 20 ng/mL GM-CSF and 10 ng/mL IL-4. On day 7, BMDCs of three groups (*n* = 4/group) were treated with DMEM (100 μL), MPLA (100 μL of 1 μg), and inactivated LBNSE (100 μL of 1 × 10^7^ FFU), respectively. (**A**) Representative gating strategy for CD11c^+^ CD86^+^ cells in BMDCs. (**B**) Representative flow cytometric plots of mature CD11c^+^ CD86^+^ BMDCs from three groups. (**C**) Statistical results of CD11c^+^ CD86^+^ BMDCs collected at 24 h post-stimulation. Data are presented as means ± SD. Asterisks indicate a significant difference between the groups at the levels of *** *p* < 0.001 and “ns” stands for “not significant”.

The gating strategy and representative flow cytometric plots of activated BMDCs are shown in Figure 1A. The representative CD11c^+^ CD86^+^ BMDCs from three groups are presented in Figure 1B. As shown in Figure 1C, in WT and TLR4^−/−^ BMDCs, inactivated-LBNSE induced significantly more maturation of BMDCs (CD11c^+^ CD86^+^ cells) than DMEM. In WT BMDCs, MPLA induced significantly more maturation of BMDCs (CD11c^+^ CD86^+^ cells) than DMEM. However, in the TLR4^−/−^ BMDCs, no significant difference in maturation of BMDCs was observed between MPLA and DMEM treatment. Together, these data suggest that MPLA promotes the maturation of BMDCs via a TLR4-dependent pathway.

### 3.2. MPLA Promotes the Maturation of cDCs in LNs Post RABV Immunization

As an adjuvant, MPLA has been licensed in Europe and the USA for human vaccines. Conventional dendritic cells (cDCs) play a crucial role in processing and presenting antigens to T cells, and these activated cDCs upregulate CD80 and/or CD86 (costimulatory molecules) or MHCII (the major histocompatibility complex class II), which could enhance the interaction between cDCs and T cells [34,35,36]. To investigate the ability of MPLA to improve the inactivated rabies vaccine in mice, we first tested the ability of MPLA as an adjuvant to promote the activation/maturation of cDCs in LNs. Three groups of C57BL/6 mice were immunized with DMEM, BPL-inactivated LBNSE and BPL-inactivated LBNSE supplemented with MPLA via intramuscular (IM) incubation, respectively. 

**Figure 2:**
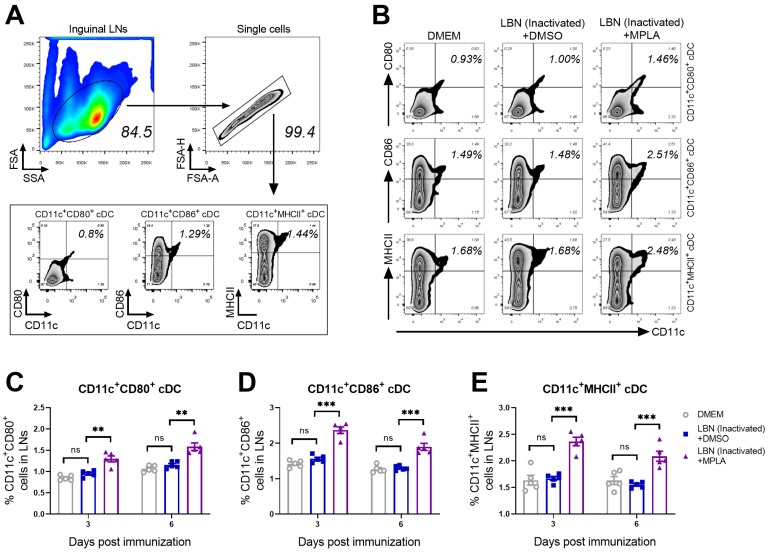
MPLA causes the activation of cDCs in inguinal LNs after RABV immunization. Three groups of C57BL/6 mice (*n* = 5/group) were inoculated with 100 μL DMEM, or inactivated LBNSE (1 × 10^7^ FFU), or the mixture of inactivated LBNSE (1 × 10^7^ FFU) and MPLA (20 μg) via IM incubation. At 7 and 14 days d.p.i., all the mice in three groups were euthanized and their inguinal LNs were collected and dispersed by grinding gently. Single-cell suspensions of the inguinal LNs were filtered through the 40-μm strainer and single cells were stained with antibodies representing the markers of CD11c^+^ CD80^+^ cells, CD11c^+^ CD86^+^ cells, and CD11c^+^ MHCII^+^ cells. Then, stained cells were analyzed by flow cytometry. (**A**) Representative gating strategy for the detection of CD11c^+^ CD80^+^ cells, CD11c^+^ CD86^+^ cells, and CD11c^+^ MHCII^+^ cells. (**B**) Representative flow cytometric plots of CD11c^+^ CD80^+^ cDCs, CD11c^+^ CD86^+^ cDCs, and CD11c^+^ MHCII^+^ cDCs cells from three groups. (**C**–**E**) Statistical results of activated **(C)** CD11c^+^ CD80^+^ cDCs, (**D**) CD11c^+^ CD86^+^ cDCs, and (**E**) CD11c^+^ MHCII^+^ cDCs are presented. Data are presented as the means ± SEM. Asterisks indicate a significant difference between the groups at the levels of ** *p* < 0.01, and *** *p* < 0.001 with “ns” standing for “not significant”.

At 7 and 14 days post-immunization (d.p.i.), the inguinal LNs of these three groups were collected and stained for detecting CD11c (a cDCs marker), CD80, CD86, and MHCII. The gating strategy and representative flow cytometric plots of the activated cDCs including CD11c^+^ CD80^+^, CD11c^+^ CD86^+^, and CD11c^+^ MHCII^+^ cDCs are shown in Figure 2A. The representative plots of activated cDCs from these three groups are analyzed and presented in Figure 2B. As shown in Figure 2C–E, the statistical analysis of activation/maturation cDCs including CD11c^+^ CD80^+^ (Figure 2C), CD11c^+^ CD80^+^ (Figure 2D) and CD11c^+^ CD80^+^ cDCs (Figure 2E) indicated that the group immunized with BPL-inactivated LBNSE supplemented with MPLA exhibited a significantly higher proportion of mature cDCs than the group immunized merely with BPL-inactivated LBNSE.

### 3.3. MPLA Enhances the Recruitment of Tfh Cells in LNs Post RABV Immunization

**Figure 3:**
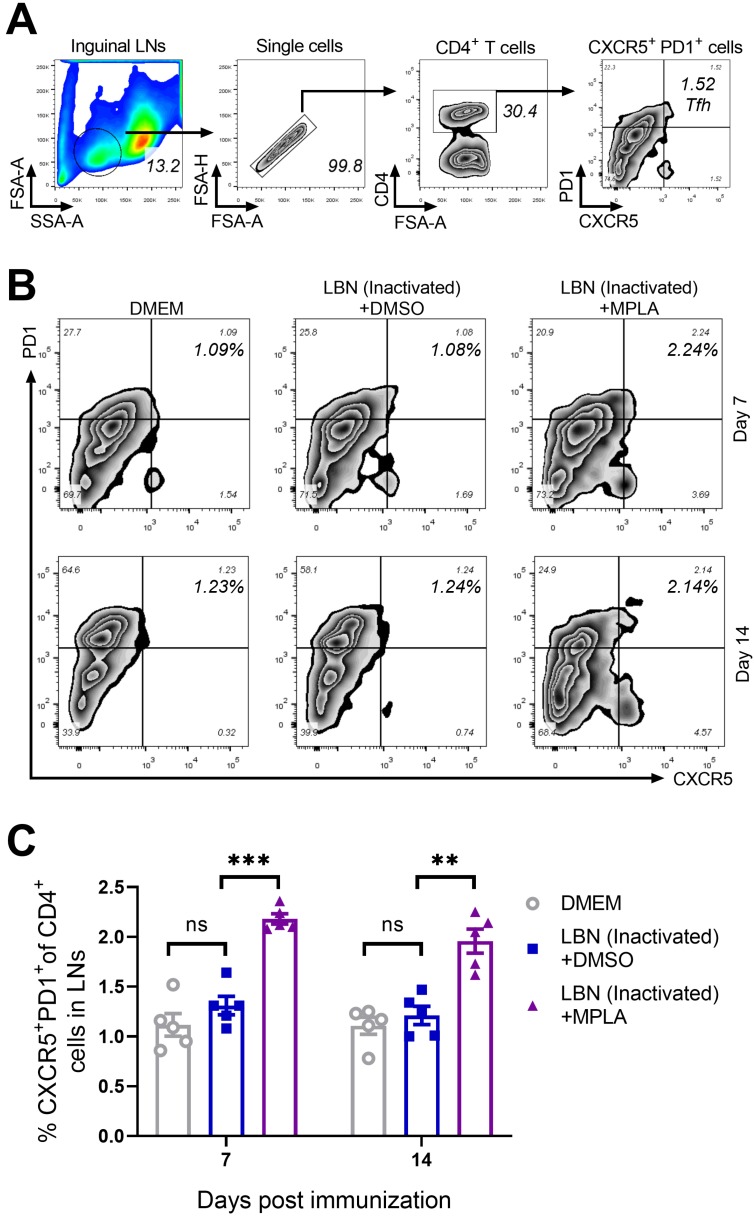
MPLA enhances the recruitment of Tfh in inguinal LNs after RABV immunization. Three groups of C57BL/6 mice (*n* = 5/group) were inoculated with 100 μL DMEM or inactivated LBNSE or a mixture of inactivated LBNSE and MPLA via IM inoculation. At 7 and 14 d.p.i., all of the mice of the three groups were euthanized, and their inguinal LNs were collected and dispersed by grinding gently. Single-cell suspensions of the inguinal LNs were filtered through a 40-μm strainer and single cells were stained with antibodies representing markers of Tfh cells. (**A**) Representative gating strategy for the detection of Tfh cells. (**B**) Representative flow cytometric plots of CD4^+^ CXCR5^+^ PD1^+^ Tfh cells from three groups. (**C**) Statistical results of CD4^+^ CXCR5^+^ PD1^+^ Tfh cells. Data are presented as means ± SEM. Asterisks indicate a significant difference between the groups at the levels of ** *p* < 0.01, and *** *p* < 0.001 with “ns” standing for “not significant”.

The costimulatory molecules CD80/CD86 expressed on antigen-presenting cells (APC), such as cDCs, enhanced the interaction between APC and T cells, and regulated the activation and recruitment of CD4^+^ T cells [15,37]. Therefore, The Tfh cells in the inguinal LNs of the group immunized with BPL-inactivated LBNSE supplemented with MPLA were investigated. The gating strategy and representative flow cytometric plots of Tfh cells (CD4^+^ CXCR5^+^ PD1^+^) are shown in Figure 3A,B, respectively. At 7 and 14 d.p.i., significantly more Tfh cells were detected in the group immunized with MPLA supplemented with BPL-inactivated LBNSE than the group immunized merely with BPL-inactivated LBNSE. However, at 7 and 14 d.p.i., no significant difference in the number of Tfh cells was observed between the group immunized with BPL-inactivated LBNSE alone and the group mock-immunized with DMEM (Figure 3C). These results indicated that the supplementation of MPLA as an adjuvant into BPL-inactivated LBNSE could facilitate the recruitment of Tfh cells in the inguinal LNs of mice.

### 3.4. MPLA Augments the Proliferation of GC B Cells in LNs Post RABV Immunization

**Figure 4:**
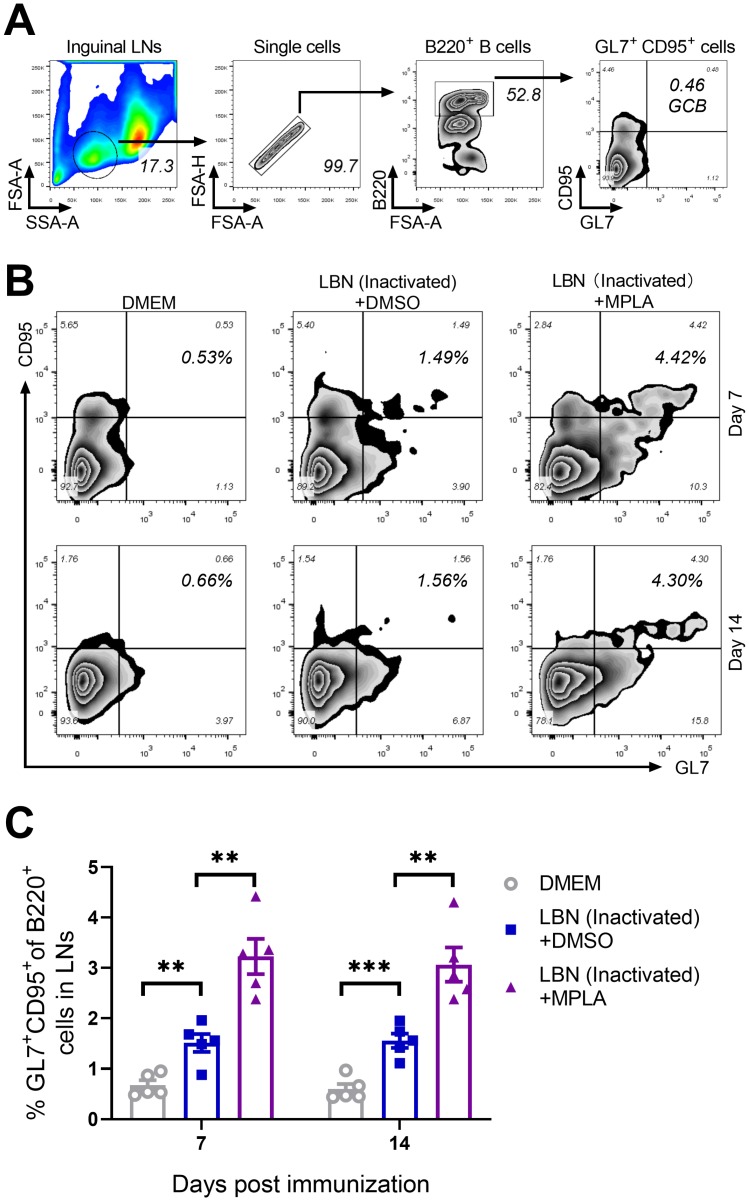
MPLA facilitates the proliferation of GC B cells in inguinal LNs after RABV immunization. Three groups of C57BL/6 mice (*n* = 5/group) were inoculated with 100 μL DMEM, or inactivated LBNSE, or the mixture of inactivated LBNSE and MPLA via IM incubation. At 7 and 14 d.p.i., all of the mice in the three groups were euthanized, and their inguinal LNs were collected and dispersed by grinding gently. Single-cell suspensions of the LNs were filtered through a 40-μm strainer and single cells were stained with antibodies representing the markers of GC B cells. (**A**) Representative gating strategy for the detection of GC B cells. (**B**) Representative flow cytometric plots of B220^+^ GL7^+^ CD95^+^ GC B cells from three groups. (**C**) Statistical results of B220^+^ GL7^+^ CD95^+^ GC B cells. Data are presented as means ± SEM. Asterisks indicate a significant difference between the groups at the levels of ** *p* < 0.01, and *** *p* < 0.001.

The quality and intensity of the GC responses are directly regulated by Tfh cells, thus providing growth and differentiation signals to GC B cells [38,39]. The effect of MPLA on the generation of GC B cells was evaluated in mice immunized with BPL-inactivated LBNSE with or without MPLA. The gating strategy and representative flow cytometric plots of GC B cells (B220^+^ GL7^+^ CD95^+^) are shown in Figure 4A,B, respectively. As expected, at 7 and 14 d.p.i., the group immunized with BPL-inactivated LBNSE supplemented with MPLA was found to generate significantly more GC B cells in the inguinal LNs than the group immunized merely with BPL-inactivated LBNSE, and the group immunized with BPL-inactivated LBNSE alone was found to generate significantly more GC B cells than the group mock-immunized with DMEM (Figure 4C). These data suggested that the MPLA as an adjuvant of inactivated rabies vaccines could promote the proliferation of GC B cells in RABV-immunized mice.

### 3.5. MPLA Facilitates the Generation of Plasma Cells (PCs) in BMs Post RABV Immunization

**Figure 5:**
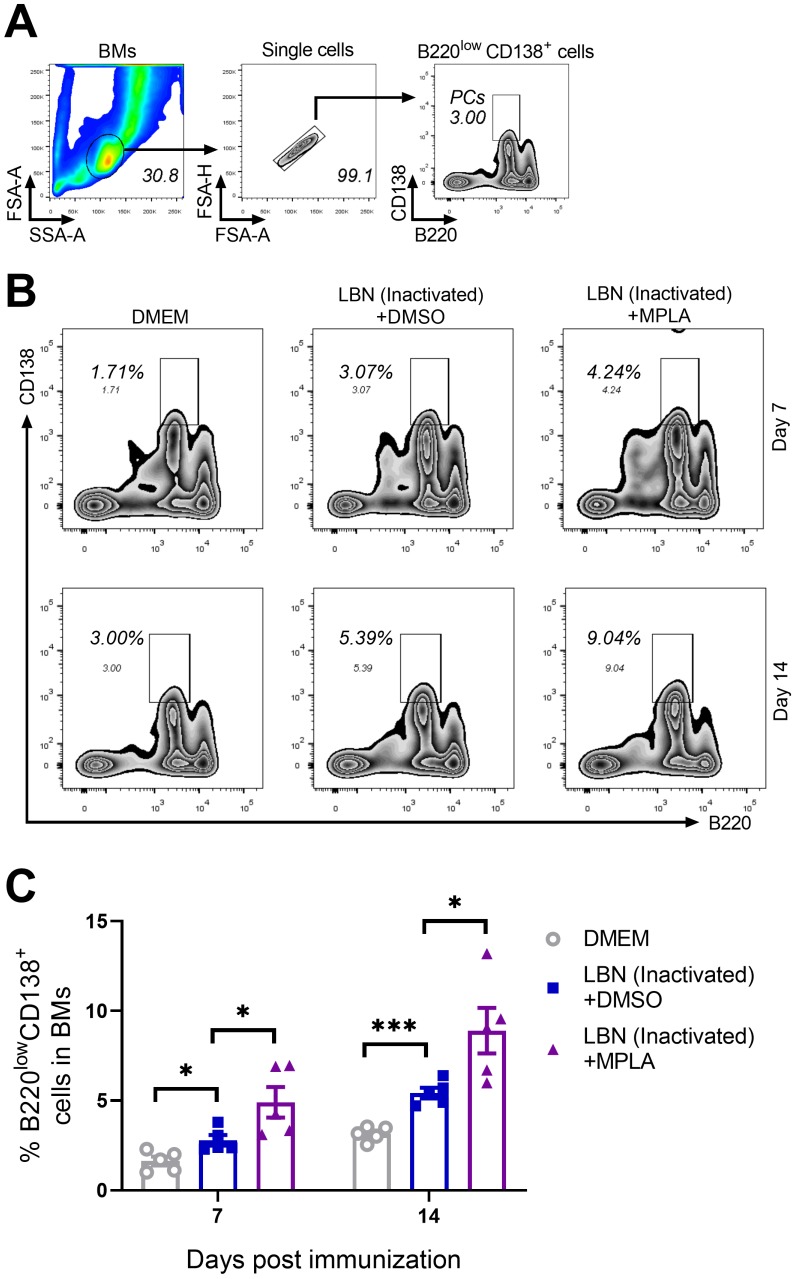
Three groups of C57BL/6 mice (*n* = 5/group) were inoculated with 100 μL DMEM, or inactivated LBNSE, or the mixture of inactivated LBNSE and MPLA via IM incubation. At 7 and 14 d.p.i., all of the mice in the three groups were euthanized, and their BM cells were collected from femurs and tibias for the detection of PCs. Single-cell suspensions of the PCs were filtered through a 40-μm strainer, and single cells were stained with antibodies representing markers of PCs. (**A**) Representative gating strategy for the detection of PC cells. (**B**) Representative flow cytometric plots of B220^low^ CD138^+^ PCs from three groups. (**C**) Statistical results of B220^low^ CD138^+^ PCs. Data are presented as means ± SEM. Asterisks indicate a significant difference between the groups at the levels of * *p* < 0.05 and *** *p* < 0.001.

The GC reaction in secondary immune organs and tissues is indispensable for the humoral immune response induced by an infection and/or immunization with antigens. Lack of GC reaction or a weak GC reaction usually causes fewer PCs and low levels of neutralizing antibodies. GC B cells can differentiate into either memory B cells or PCs secreting large quantities of antibodies in response to antigens [40,41]. Hence, the BMs of mice immunized with BPL-inactivated LBNSE added with or without MPLA were collected at 7 and 14 d.p.i. and were analyzed for PCs by flow cytometry. The gating strategy and representative flow cytometric plots of PCs (B220^low^ CD138^+^) are shown in Figure 5A,B, respectively. At both 7 and 14 d.p.i, the group immunized with MPLA-supplemented BPL-inactivated LBNSE generated significantly more PCs in the BM than the group immunized with BPL-inactivated LBNSE alone, and the group immunized with BPL-inactivated LBNSE alone generated significantly more PCs than the group mock-immunized with DMEM (Figure 5C). These data suggested that the MPLA as an adjuvant of inactivated rabies vaccines could promote the generation of PCs in immunized mice.

### 3.6. MPLA Improves Antibody Production and Protection Against Virulent RABV Challenge

**Figure 6:**
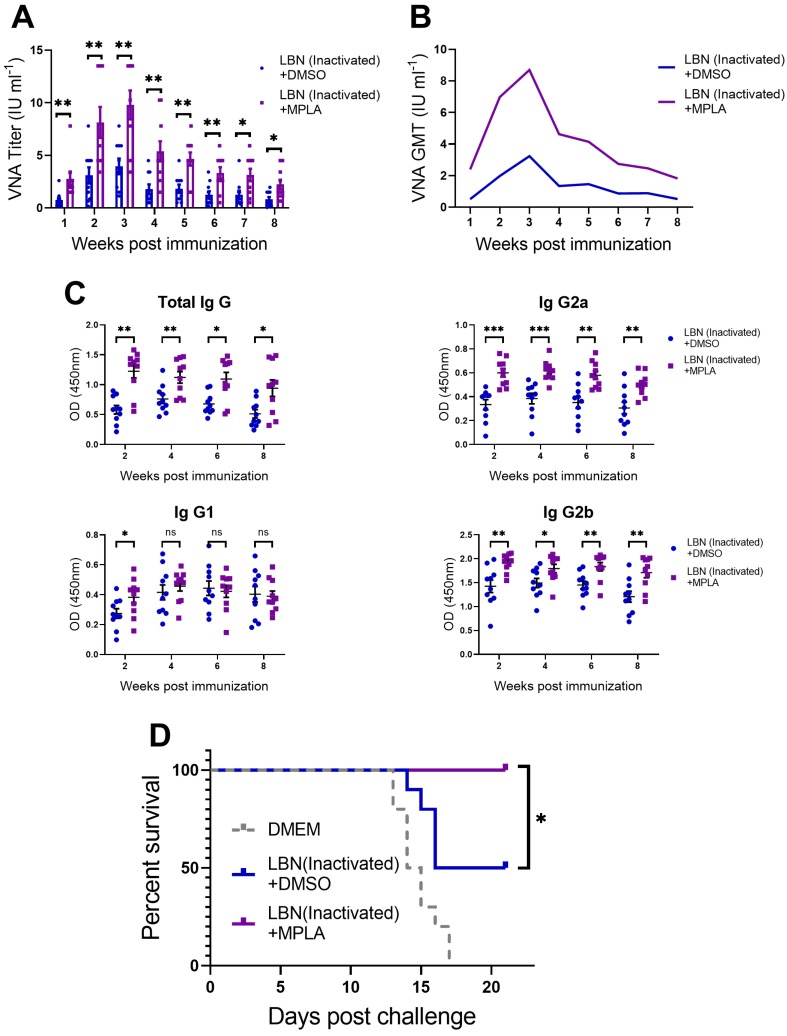
Three groups of C57BL/6 mice (*n* = 5 /group) were inoculated with 100 μL DMEM, or inactivated LBNSE, or a mixture of inactivated LBNSE and MPLA via IM incubation. The serum of the mice was harvested every week until the eighth-week post-immunization (p.i.). (**A**) Sera of mice (1 to 8 weeks) were tested for VNA against RABV. (**B**) The geometric mean of VNA was analyzed and presented. (**C**) RABV-specific total-IgG, IgG1, IgG2a and IgG2b in the sera of mice (week 2, 4, 6 and 8) were tested by indirect ELISA. (**D**) At 8 weeks p.i., all of the mice in the three groups (*n* = 10/group) were challenged via IM incubation with 100 × LD_50_ of DRV-Mexico, and the mortality ratio was then monitored for 21 days. Data are presented as means ± SEM. Asterisks indicate significant difference between the groups at the levels of * *p* < 0.05, ** *p* < 0.01, and *** *p* < 0.001 with “ns” standing for “not significant”.

As is well known, humoral immune responses give rise to the production of RABV-specific VNA. VNA interferes with the RABV virion binding to a receptor and plays a pivotal role in blocking viral fusion with the cells. In order to evaluate the humoral immune responses induced by inactivated RABVs in mice, we immunized three groups of ICR mice (*n* = 10/group) via IM incubation with 100 μL of inactivated LBNSE (1 × 10^7^ FFU) alone, or a mixture of inactivated LBNSE (1 × 10^7^ FFU) and MPLA (20 μg), or with DMEM, respectively. The VNA titers were determined by the FAVN method. As shown in Figure 6A,B, a significantly higher VNA level was observed in the MPLA-supplemented LBNSE-inactivated immunized group than in the group immunized with inactivated LBNSE alone at all indicated points. At 1 and 8 weeks post-immunization (p.i.), only 5 out of 10 mice in the group immunized with inactivated LBNSE alone exhibited VNA levels ≥ 0.5 IU/mL, while all 10 mice in the group immunized with the mixture of inactivated LBNSE and MPLA displayed levels ≥ 0.5 IU/mL. Additionally, RABV-specific total-IgG, IgG1, IgG2a and IgG2b in the serum of immunized mice were determined with the above-mentioned method. As shown in Figure 6C, the mixture of inactivated LBNSE and MPLA induced significantly higher total-IgG, IgG2a, and IgG2b at all indicated points. However, only in the first week, the mixture of inactivated LBNSE and MPLA induced significantly higher IgG1. 

Hence, at 8 weeks p.i, all of the mice (*n* = 10/group) were challenged with 100 × LD_50_ of DRV-Mexico via IM incubation, and the mortality rate was then monitored for 21 days. As shown in Figure 6D, all of the mice in the mock-immunized group succumbed to rabies within 17 days, while 100% of the mice immunized with MPLA-supplemented inactivated LBNSE were protected from the virulent RABV challenge, compared with only 50% survival of the mice in the group immunized with inactivated LBNSE alone.

## 4. Discussion

Humoral immunity plays a primary role in preventing RABV infection, and it can be initiated by the cDCs residing in secondary lymph organs. Our previous studies have reported that live rabies vaccines achieved a better humoral immune response effect through co-expression of viral carriers and DC activators including the macrophage inflammatory protein 1α (MIP1α) [31], GM-CSF [22] and HMGB1 [19], and that the T cell-dependent B cell response could be initiated by DC activation. However, due to the safety issue, these live rabies vaccines are restricted only to wild animals and inactivated rabies vaccines are still widely used in human and domestic animals. Considering the relatively low efficiency of inactivated rabies vaccines, supplementation with adjuvants stimulating the maturation of cDCs is a promising strategy to enhance the immunogenicity of inactivated rabies vaccines. 

Most TLR-related agonists are potential candidates for rabies vaccine adjuvants due to their ability to induce a pro-inflammatory cytokine response and immune cell activation. RABV-derived lipopeptide CE536 conjugated to a TLR7 agonist (imiquimod) was reported to have improved DC phenotypic maturation and the Th1-biased humoral immune response in mice [42]. The TLR3 agonist PIKA (a chemical analog of double-stranded RNA) as an adjuvant of the rabies vaccine enhanced both humoral and cellular immunity [14]. The synthetic TLR9 agonist IMO-2170 increased the magnitude of the humoral immune response induced by the rabies vaccine [12]. The synthetic TLR4 agonist glucopyranosyl lipid A (GLA-SE) as an adjuvant increased the protection provided by the inactivated RABV-based Ebola vaccine [43]. In this study, we found that the TLR4 agonist MPLA is a promising adjuvant for the inactivated rabies vaccine. 

TLR4 recognizes both microbial and endogenous ligands, and is a relatively promising immune sensor [17,18,44]. MPLA is a TLR4 agonist and is derived from the product of chemically modified lipooligosaccharide with a diminished toxic pro-inflammatory effect and adequate immunogenicity. Recently, MPLA has been licensed in Europe and the USA for human vaccines [45,46]. A previous study reported that MPLA as an adjuvant activated CD3^+^ CD4^+^ and CD3^+^ CD8^+^ T cells, and it might provide early protection against RABV by accelerating antibody production [46]. Our results confirmed the role of MPLA in activating immune cells (such as BMDC and cDC) and revealed that MPLA promoted the activation/maturation of BMDC in vitro through the TLR4 pathway and the activation/maturation of cDCs in the inguinal LNs of immunized mice. Moreover, the highly expressed costimulatory molecules CD80/CD86 on the surface of DCs is necessary for T cell activation and survival. The activated-DC-secreted cytokines including IFN-γ and IL-12 were reported to enhance the T-dependent B cell response and antibody level [16,47]. Our results revealed that the recruitment of Tfh, and the proliferation of GC B cells and PCs following the maturation of cDC in vivo all improved after supplementing MPLA into inactivated rabies vaccines. Therefore, MPLA serving as an adjuvant could improve the T-dependent B cell response after immunization.

The absence of or low level of VNA in animals or humans immunized with inactivated rabies vaccines make them susceptible to virulent RABV challenge. Useful adjuvant application or multistep injections of inactivated rabies vaccines could provide enough protection against RABV [48,49]. The World Health Organization (WHO) has recommended a relatively conservative level of 0.5 IU/mL VNA titer for human vaccination [49,50]. Our VNA test results indicated that all of the mice in the group immunized with MPLA-supplemented inactivated rabies vaccines exhibited a VNA titer ≥ 0.5 IU/mL at 1 and 8 weeks p.i., showing the accelerative and lasting effect of MPLA on the humoral immune response. Moreover, our virus challenge test result indicated that MPLA could provide improved protection against a virulent RABV challenge when all mice have received pre-exposure (PrEP) vaccinations. Previous studies reported that MPLA could induce a strong Th1 response, which was associated with the induction of IgG2a and IgG2b antibodies in mice [20,21]. The Th1 response was associated with both the production of VNA and the clearance of wild type RABV from the central nervous system (CNS) [51]. The RABV-specific IgG isotyping test revealed that MPLA-supplemented inactivated rabies vaccines mainly induced Ig class switching to IgG2a and IgG2b, which implied a beneficial effect of adjuvant MPLA on the inactivated rabies vaccine.

In summary, this study demonstrates that MPLA as an adjuvant of inactivated rabies vaccines could facilitate the maturation of DCs, and effectively promote the proliferation of Tfh cells, GC B cells, and PC cells, thus significantly enhancing RABV-specific VNA production and improving the protection against a virulent RABV challenge.
